# Supplementary material for: Inverse mapping of quantum properties to structures for chemical space of small organic molecules
Source: Nat Commun. 2024 Jul 18;15:6061. doi: 10.1038/s41467-024-50401-1 (PMC11258234; doi:10.1038/s41467-024-50401-1)
Supplement: Supplementary file 1 — Supplementary Information [file 41467_2024_50401_MOESM1_ESM.pdf]

Supplementary Information (SI) for:

Inverse Mapping of Quantum Properties to Structures for Chemical Space of Small Organic Molecules

Alessio Fallani,[1] Leonardo Medrano Sandonas,[1,2] and Alexandre Tkatchenko[1]

[1] *Department of Physics and Materials Science, University of Luxembourg, L-1511 Luxembourg City, Luxembourg.*

[2] *Institute for Materials Science and Max Bergmann Center of Biomaterials, TU Dresden, 01062 Dresden, Germany.*

[*] Corresponding authors: Alessio Fallani (`alessio.fallani.001@student.uni.lu`), Leonardo Medrano Sandonas (`leonardo.medrano@tu-dresden.de`), Alexandre Tkatchenko (`alexandre.tkatchenko@uni.lu`)

Supplementary Note 1. Retrieval of 3D molecular representation and structure

Molecular reconstruction procedure

Given our implementation, a fixed input and output dimensionality is needed. We decided to pad the Coulomb matrices (CM) of QM7-X molecules by setting a standard format for the composition. Namely, we checked the maximum number of atoms in a single molecule per atom type, and then the concatenated set of these constitutes the standard format for the Coulomb matrix. Indeed, we have that the standardized format for these molecules is $\{O, O, N, N, N, C, C, C, C, C, C, C\}$. The entries corresponding to the atoms within the molecule are then filled with the Coulomb matrix values, while, for the other entries, the corresponding rows and columns are filled with zeros. Notice that, with this choice of padding, it is in principle possible for the model to generate molecules with more heavy atoms than the biggest molecule in QM7-X dataset.

For what concerns the retrieval of cartesian coordinates, as mentioned in the main text, we can easily retrieve atomic compositions and distance matrices from the CM representation. The procedure for retrieving cartesian coordinates then follows a multidimensional scaling (MDS) method well known in Euclidian distance matrices (EDMs) theory[1]. Thus, given the distance matrix $D_{ij} = ||\mathbf{x}_i - \mathbf{x}_j||$, we can first define:

$$\begin{aligned} M_{ij} &= \frac{D_{1j}^2 + D_{i1}^2 - D_{ij}^2}{2} \\ &= (\mathbf{x}_i - \mathbf{x}_0) \cdot (\mathbf{x}_j - \mathbf{x}_0) \\ &= \tilde{\mathbf{X}}\tilde{\mathbf{X}}^T, \end{aligned}$$

and posteriorly diagonalize it to obtain $M = USU^T$, from which we get $\mathbf{X} = U\sqrt{S}$. In the case in which D is a valid EDM, the diagonalization will yield only 3 eigenvalues different from zero, as the final X will have dimension $3 \times N$ (with N as the number of entries of the EDM). In our case, all possible outputs of the model can be considered as valid Coulomb matrices, hence, valid EDMs. This problem has been addressed in Ref.[2] by enforcing terms in the loss function to bias the model to automatically generate valid

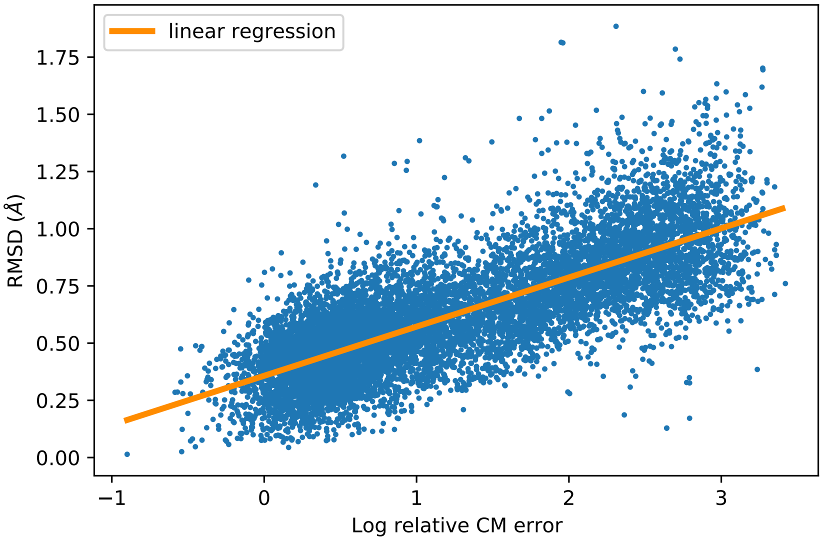

Supplementary Figure 1 Correlation plot and linear interpolation between the logarithm of the relative error on CM reconstruction and the corresponding RMSD.

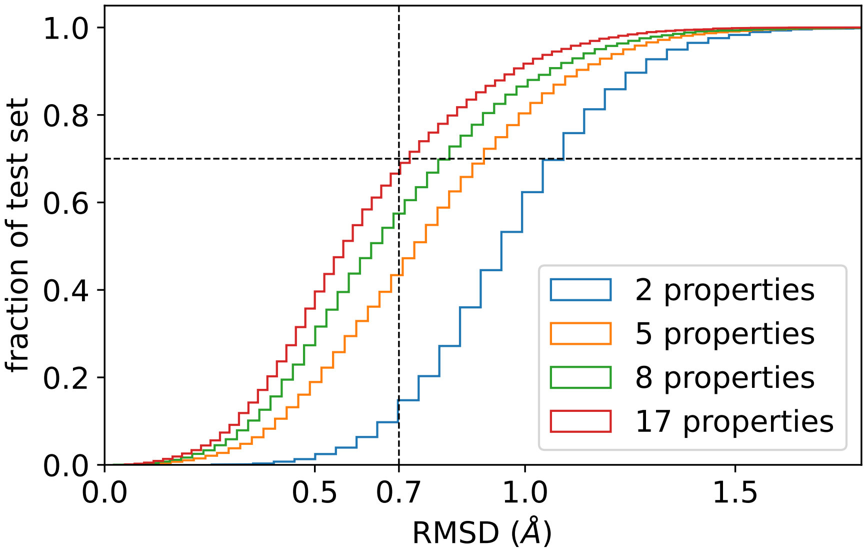

Supplementary Figure 2 Cumulative distribution function for RMSD computed using molecules from the test and different numbers of QM properties in the reconstruction procedure.

EDMs. We instead decided to take a simpler approach by only keeping the eigenvectors associated to the highest eigenvalues of the M matrix, effectively approximating the output to the closest valid EDM (hence the closest valid CM). Despite not being a very refined method, this turned out to be good enough for our proof-of-concept implementation.

As the procedure to reconstruct the 3D molecular structure from a CM is not linear and involves a truncation to the closest valid EDM, it is also interesting to look at how the RMSD correlates with the error on representation reconstruction. In Suppl. Fig. 1, one can observe that a bulk linear correlation is present between RMSD and the logarithm of the relative error on the CM.

Analysis of the reconstruction performance: property type and RMSD threshold

Here, we analyze the influence of properties in the reconstruction performance. In Suppl. Fig. 2, we show the RMSD cumulative distribution from the test set for different number of QM properties used in the training procedure. Considering that the threshold for reasonable reconstruction is approximately 0.7 Å, we observed that, although the error in the representation appears to plateau after 7 or 8 properties, it is advantageous to retain all 17 properties. The threshold of 0.7 Å is an empirical value based on how well the model reconstructs molecular topology, as discussed in the main text. We found that there is a general deterioration in the reconstruction of the topology of molecular structures as well as broken molecules after this threshold. In Suppl. Fig. 3(a), we quantify this effect by plotting the percentage of molecules with correctly reconstructed topology with RMSD values below a given threshold. We consider a molecule to have a correctly reconstructed topology if there is a complete match in the adjacency matrices of the reconstructed and original molecule (only heavy atoms backbone), *i.e.*, the number of topological errors in the molecule is zero. To compute this number per molecule, we first optimize the overlap and atom permutation between original and reconstructed molecule by iteratively using quaternion optimization for overlap in 3D space and Hungarian algorithm for reordering atoms (necessary as different orders in subsets of same atom type can lead to wrong 3D space overlap). Then, using OpenBabel, we extract the adjacency matrix for both the original and reconstructed molecules. Afterward, we sum up the differences in absolute value divided by the

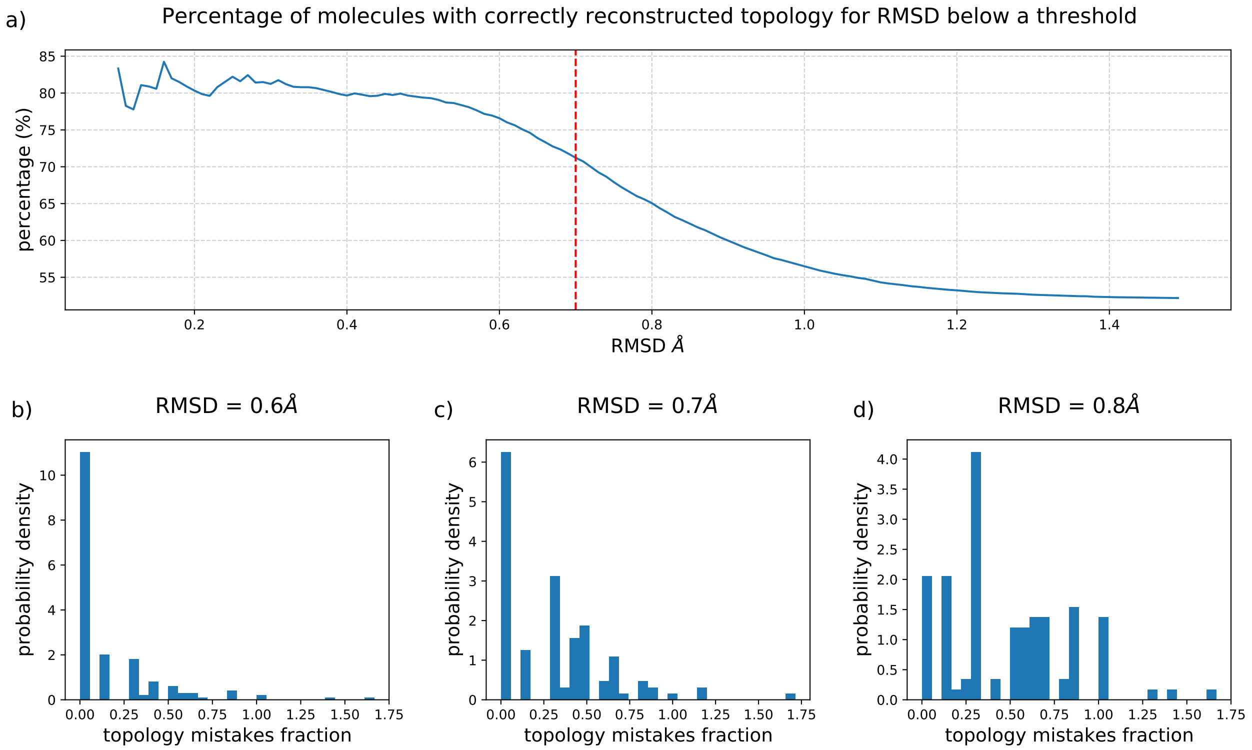

Supplementary Figure 3 a) Percentage of reconstructed molecules with correct topology up to a given RMSD value (blue curve), *e.g.*, when considering molecules with RMSD < 0.4 Å then 80% of those are reconstructed with correct topology. Also a vertical red line is reported at 0.7 Å where we picked the threshold. Distributions of the topological errors fraction (correct bonds over the total number of bonds in the molecule) for RMSD = b) 0.6 Å, c) 0.7 Å, d) 0.8 Å.

sum of the elements of the original adjacency matrix. While the initial noisy behavior in Suppl. Fig. 3(a) is due to the small number of molecules for low RMSD values, we see a slope that seems to change inflection around RMSD=0.7 Å. This is further analyzed by looking at the distributions of relative topological errors per molecule for fixed values of RMSD in panels (b), (c) and (d) of Suppl. Fig. 3. To make these plots, we select the molecules within a ± 0.05Å range of a given reference RMSD value, consequently, the molecules will fall respectively in the intervals $[0.55, 0.65)$Å, $[0.65, 0.75)$Å, $[0.75, 0.85)$Å.

Furthermore, we investigated the effect in the performance model of considering extensive and intensive properties separately in the training procedure. In Suppl. Fig. 4, we show the RMSD histogram and its corresponding cumulative function for the models trained using the set of 17 QM properties, only extensive properties, and only intensive ones. These results verify the need to use both types of properties in the training of our final model to have a better reconstruction of molecular structures. The models trained on separated properties present a higher number of structures with large RMSD compared to the final model, indicating a lower performance in structure reconstruction. Moreover, the model trained only on intensive properties was able to reconstruct with the right chemical composition approximately 8,000 from 10,000 molecules of the test set, see Suppl. Fig. 4(b). This decrease from 99.96% to 75.85% in molecular reconstruction comes together with an increment of the $\langle RMSD \rangle$ up to 0.7 Å as well as a reduction up to 55% of tested molecules reconstructed with an RMSD below 0.7 Å.

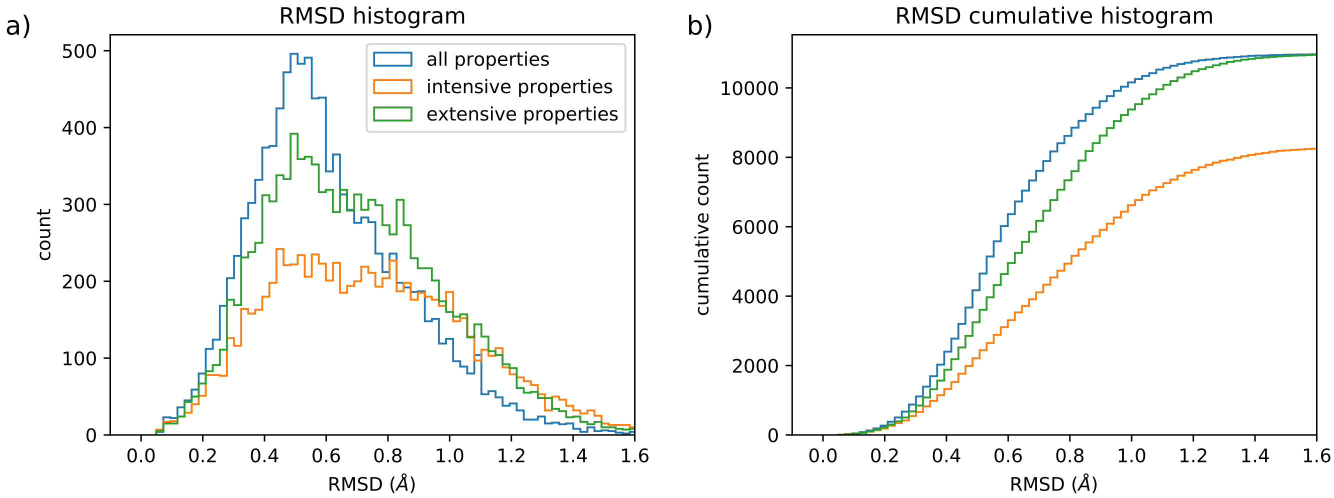

Supplementary Figure 4 a) Histograms and b) cumulative distribution function of the RMSD for molecules from the test set in molecular reconstruction evaluation when considering all properties, only intensive properties, and only extensive properties in the training procedure. In either case, the counts are scaled to show the difference in performance for composition reconstruction.

Supplementary Note 2. t-SNE representation of latent space structure

To strengthen our findings regarding the correlation between QM properties and the structure of the 2D principal component analysis (PCA) of the latent space of the VAE encoder (see Fig. 3 of the main text), we conducted the same analysis by using t-distributed stochastic neighbor embedding (t-SNE)[3]. This technique is widely used in the field of chemoinformatics for dimensionality reduction and visualization of high-dimensional data. Accordingly, we have found that the t-SNE representation of this latent space displays the same behavior as observed with PCA, *i.e.*, properties with a higher value of gradient attribution map A (thus, more relevant) are qualitatively better at organizing the latent space. Selected results for PCA and t-SNE are shown in Suppl. Fig. 5 and Suppl. Fig. 6, respectively. The coloring of the plots corresponds to the properties in the order derived from the gradient attribution map. Indeed, the most-well organized projections are determined by properties such as exchange energy E_{XX} and kinetic enegy E_{KIN} which exhibit the highest A values. Contrarily, the projections associated with properties such as the dipole moment ζ and HOMO-LUMO gap E_{GAP}, characterized by the lowest A value, are the least correlated. Moreover, we have found an overlap between the t-SNE representation of both the latent space representation from the VAE (encoding of reconstructed CMs) and property encoder (see Suppl. Fig. 7), as it as obtained with PCA (see Fig. 5 of the main text). These results demonstrate that the insights about the structure of the latent space remain consistent irrespective of the dimensionality reduction technique employed.

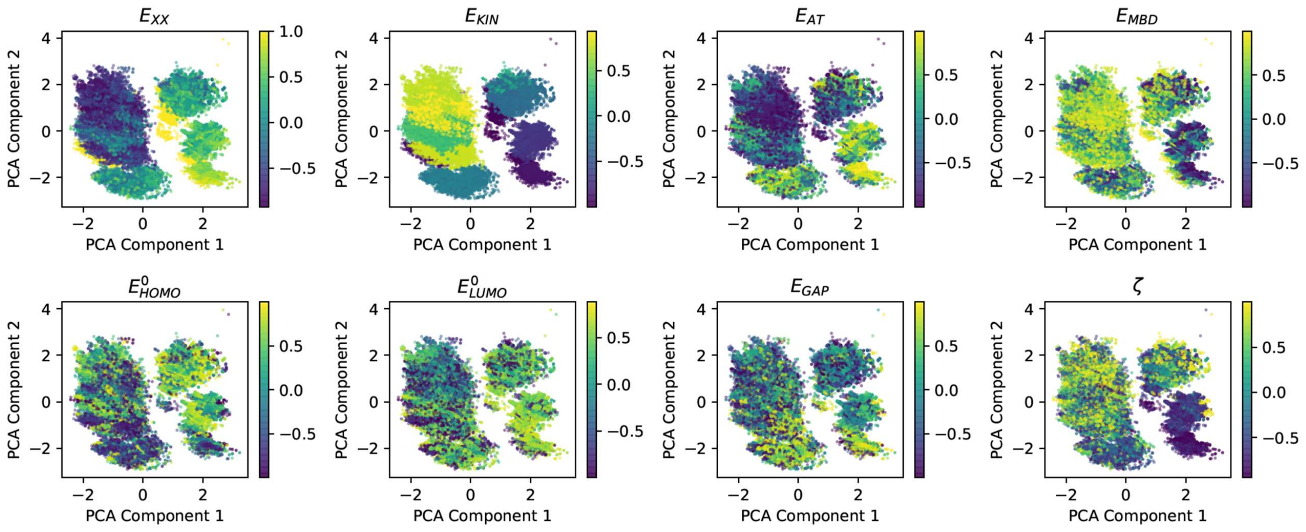

Supplementary Figure 5 PCA representation of the latent space of the QIM model colored with different properties. For the colorscheme we rescaled the properties and applied a hyperbolic tangent to avoid obscuration effects from outliers. The properties are ordered following the relevance criterion based on the gradient attribution map A outlined in Fig. 3 of the main text, *i.e.*, E_{XX} and ζ present the highest and lowest A values.

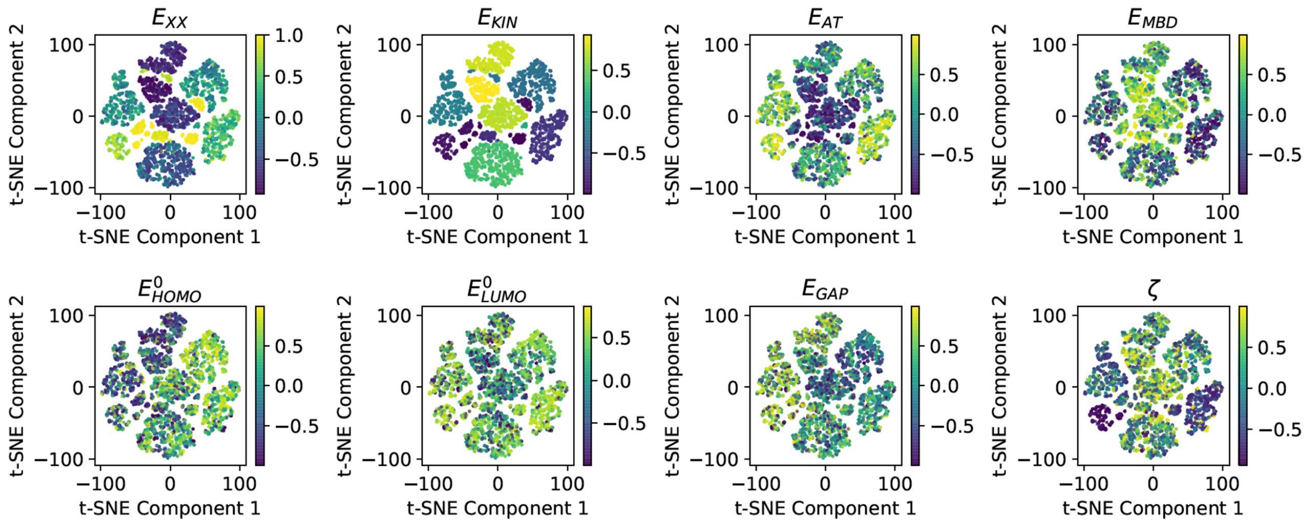

Supplementary Figure 6 t-SNE representation of the latent space of the QIM model colored with different properties. For the colorscheme we rescaled the properties and applied a hyperbolic tangent to avoid obscuration effects from outliers. The properties are ordered following the relevance criterion based on the gradient attribution map A outlined in Fig. 3 of the main text, *i.e.*, E_{XX} and ζ present the highest and lowest A values..

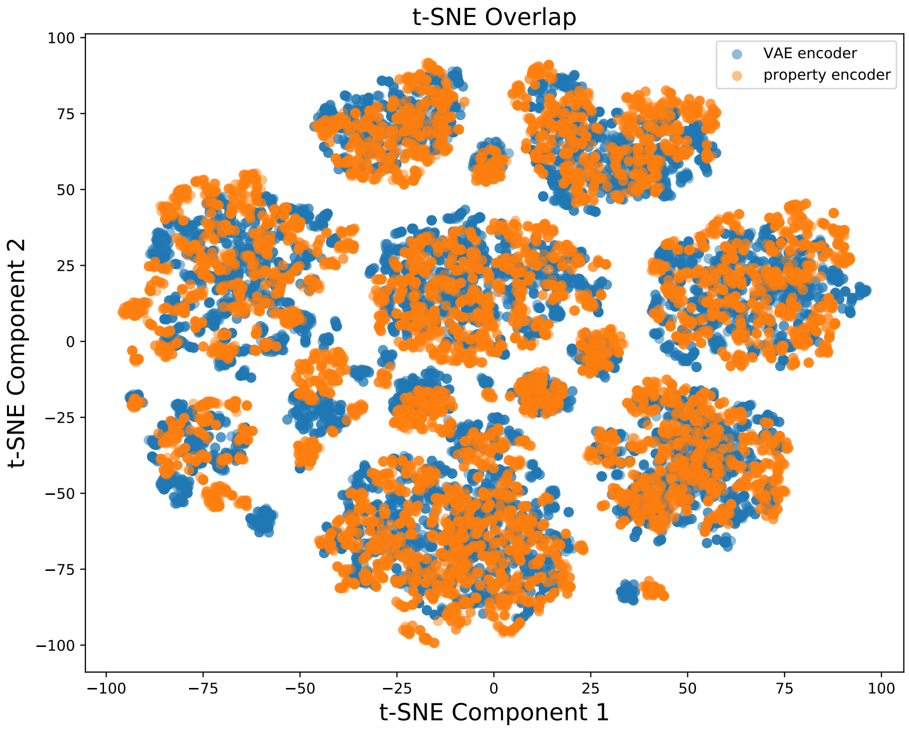

Supplementary Figure 7 Overlap of t-SNE components between both the latent space representation from the VAE and the property encoders.

Supplementary Note 3. Implementation details

Architecture and training

Here, we provide a detailed explanation of the architecture used for this implementation, as well as the training hyperparameters and schedules. The neural networks used in both the VAE and the property encoder are feed-forward neural networks with the batch norm and a dropout layer with $p = 0.1$ applied after every hidden layer. The VAE encoder is composed of one larger network outputting the mean of the latent distribution, namely made of $\{\textit{input size}, 2048, 512, \textit{latent size}\}$ neurons with swish activation, and a smaller network for the variance made of $\{\textit{input size}, 256, \textit{latent size}\}$ neurons with hyperbolic tangent activation. As for the decoder network, it has a mirrored structure to the encoder. The property encoder instead is composed of three different modules. A network made of $\{\textit{input size}, 128, 128, 32 - \textit{input size}\}$ neurons with hyperbolic tangent activation is used as an input enhancing feature, and its output is then concatenated with the set of properties. This is equivalent to a skip connection layer which is then the input shared by the two networks for the mean and variance of the latent distribution. These are respectively made of $\{32, 2048, 1024, 1024, \textit{latent size}\}$ neurons with swish activation and of $\{32, 128, \textit{latent size}\}$ neurons with hyperbolic tangent activation. The latent space used for this implementation is 21 dimensional, while the standardized size of Coulomb Matrix is 78 and the number of properties used is 17. Note that as we are autoencoding the Coulomb matrix it is not a problem for the property dimension to be lower than the latent space dimension. As for what concerns the loss function (see Eq.(2) in the main text), the β parameter follows the exponential reduction schedule $\beta = 3\gamma^{epoch}$ with $\gamma = 0.995$ up to the limit value of $\beta = 1$. The τ parameter is instead set to a fixed value $\tau = 2$ throughout training. The learning rate is initialized at 10^{-3} and then follows a plateau reduction learning rate schedule with 25 steps of patience and a reduction factor of 0.9. Training is stopped at a learning rate convergence value of 10^{-6}. The training has been performed using one Tesla P100 GPU.

Motivating Beta scheduling and hyperparameters

Considering the framework of our proposed implementation, it have to consider that, throughout the training process, we will observe the following effects on competition

- **Posterior collapse decreases latent predictor loss:** Since predicting a value that does not change too much when altering the input is easier than predicting one that undergoes significant changes, having a latent representation that is too close to the uninformative prio $\mathcal{N}(0, \mathbb{I})$ will decrease the loss for the latent space prediction task. In turn, this will also hinder the reconstruction performance and the quality of the property-to-structure mapping as the latent representation will not be expressive enough.

- **Scattered latent space decreases reconstruction loss but increases latent predictor loss:** On the other hand, a more expressive latent space (not collapsed on the prior) would make it easier for the decoder to reconstruct the molecule. However, this complicates the task of the latent space predictor, making it more challenging to accurately deduce the latent representation.

To strike a balance between these effects, we introduced the ad-hoc scheduling described in the previous section for the parameter β. The idea behind this scheduling is that in the early stages of training, we want to obtain an initially compact representation, which makes a good starting point for the training of the VAE component of the implementation, but also makes it initially easy for the latent space predictor to have a low loss. After this initial phase, we want the latent space to start being more expressive. This should happen slowly enough so that the latent space predictor can keep up with the changes in the internal representation of the VAE. Meanwhile, for the VAE decoder, it will be increasingly easier to reconstruct the original molecule.

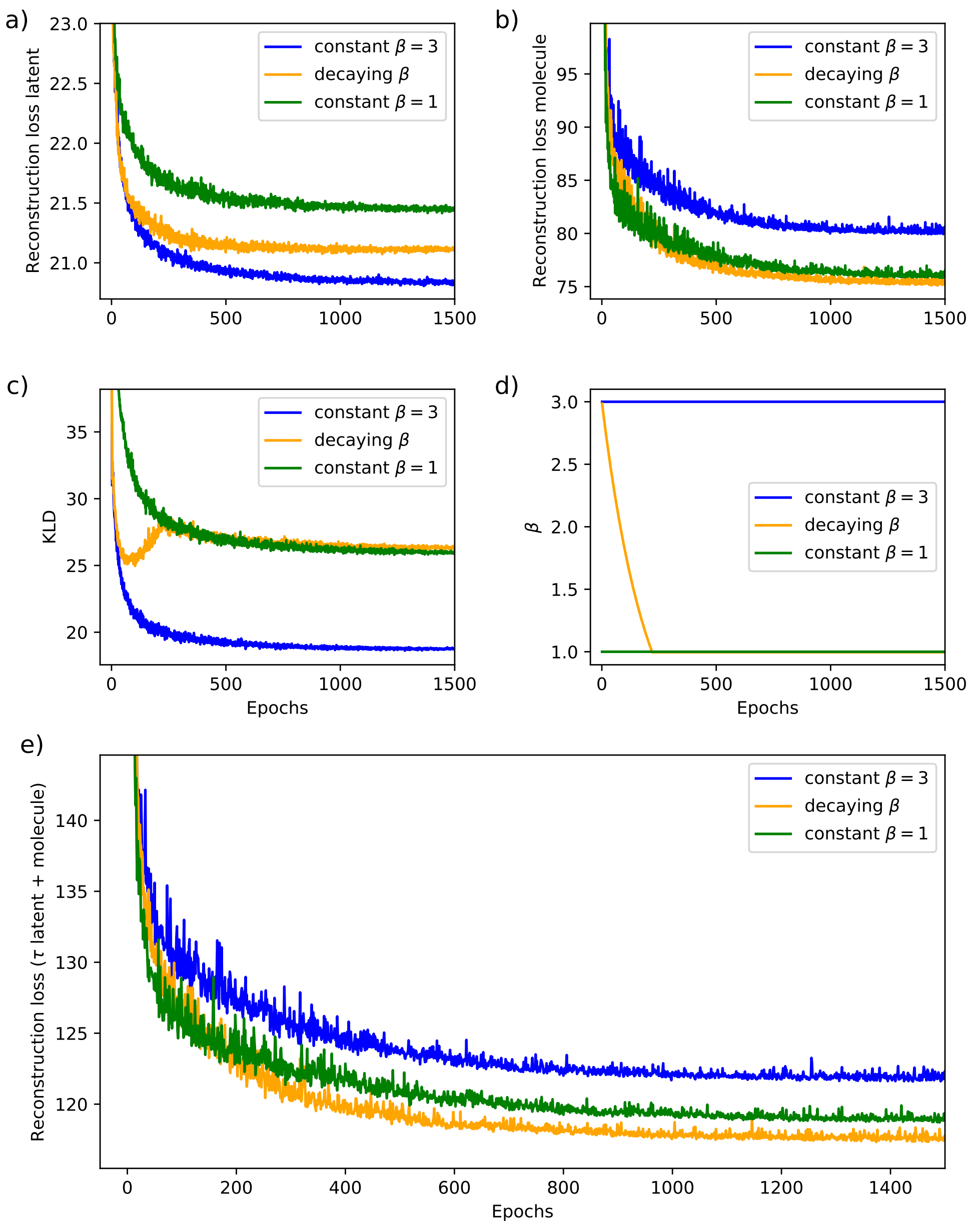

Supplementary Figure 8 Variation of loss components during the training process considering different scheduling of the β parameter. a) Reconstruction loss latent, b) reconstruction loss molecule, c) KLD component, d) β parameter, and e) total reconstruction loss.

In Suppl. Fig. 8, we report the comparison of the behavior of the loss components together with three different scheduling for the β parameter, namely $\{\beta = 3, \beta = 1, \beta = 3\gamma^{epoch}\}$ with $\gamma = 0.995$. Starting from $beta = 3$, we can observe that the KLD component of the loss is minimized, implying a more compact latent space, while the reconstruction loss of the latent representation is low (as expected from the first earlier considered effect). This finding, along with a higher value of the molecule reconstruction loss, indicates a less expressive latent representation. For $\beta = 1$, it can be observed from the KLD that the latent space never compactifies as much as in the previous case, leading to a worse performance in the latent space predictor. The scheduling with decaying β provides instead, as we wanted, an initial phase of latent space compression, where latent reconstruction loss is still as low as in the $beta = 3$ case. Then, we see an increase of the KLD

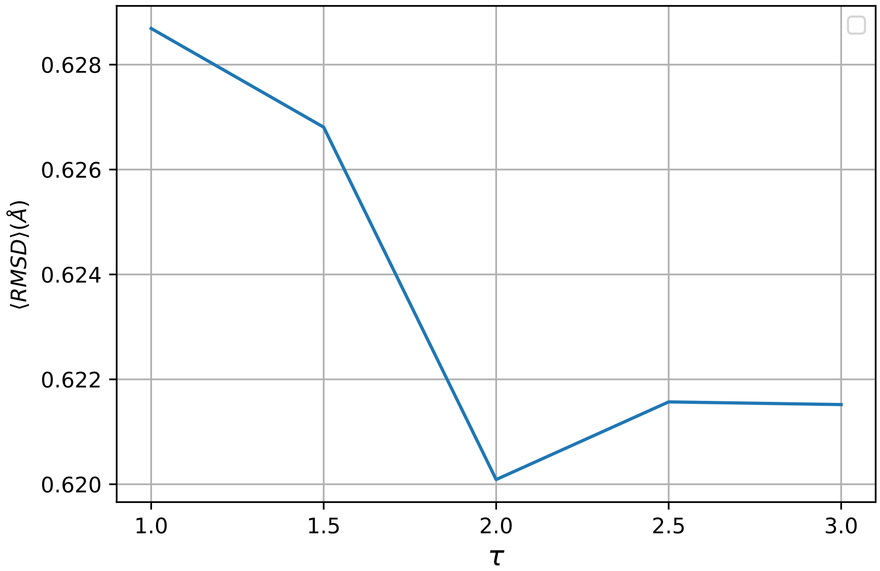

Supplementary Figure 9 ⟨RMSD⟩ obtained by the trained model with different τ values. We have used a fixed number of 1000 epochs to train these models.

which, from being lower than the plateau from the $\beta = 1$ case in the first phase, goes back up and plateaus close to the value for $\beta = 1$. For the molecule reconstruction loss, the use of this scheduling results in a more expressive latent representation that has learned to capture relevant features during the compression phase. Moreover, the higher value of KLD is a confirmation that the low loss in latent space reconstruction does not come from a collapse of the internal representation towards the uninformative prior $\mathcal{N}(0, \mathbb{I})$. Suppl. Fig. 8(e) shows a summary of these results by considering only the reconstruction components of the loss for all the considered schedules. Finally, regarding the choice of the τ parameter, we experimented with various values within the range of $(1, 3)$. This task led us to the determination that $\tau = 2$ was the optimal choice, despite observing minimal impact on the final performances in terms of $\langle RMSD \rangle$, see Suppl. Fig. 9.

Supplementary Note 4. Multivariate Gaussian distributions for fitting the property space

The method we employed to determine the number of multivariate Gaussians used for fitting the property space is the Bayesian Information Criterion (BIC). Following this method, we have to minimize the quantity:

$$BIC = -2\log(p(x|\hat{\theta})) + k\log(n),$$

where $\hat{\theta}$ is the set of parameters identifying the maximum likelihood multi-Gaussian model for a fixed number of multivariate Gaussians, $p(x|\hat{\theta})$ is the probability that the datapoints x are sampled from the corresponding model, k is the number of degrees of freedom in the model, and n is the number of samples. The idea of the criterion is to select the model structure that maximizes the likelihood term while penalizing overparameterization with the other term. We tested all numbers of Gaussians from 1 to 100, see the results for the last 30 cases in Suppl. Fig. 10. Here, it was found that 91 is indeed the best value for our fitting problem.

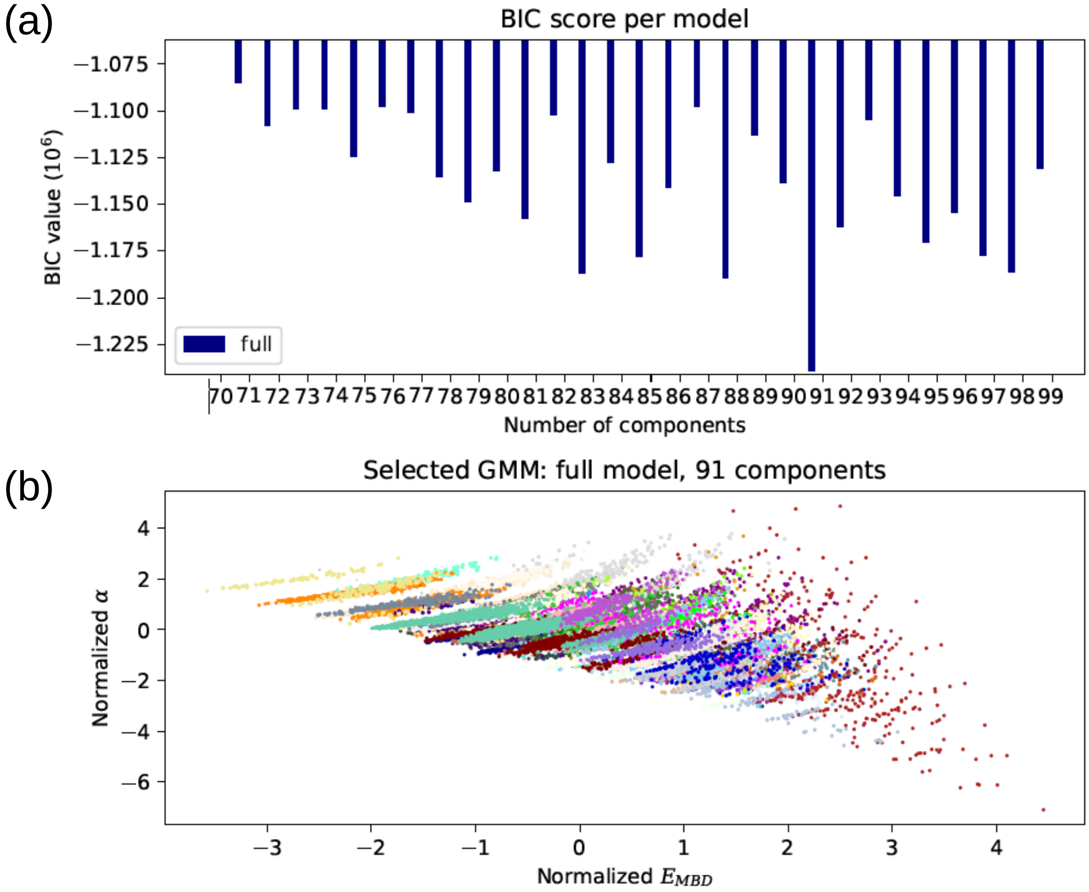

Supplementary Figure 10 (a) BIC score for models with number of Gaussians going from 70 to 100. (b) Scatter plot of the (E_{MBD}, α)-space for QM7-X molecules, we assigned a different color for each group of point assigned to a different gaussian distribution from the model (91 distributions).

Supplementary Note 5. Generation of molecules with targeted QM properties

Extension of the analysis

Given that the precision of the model depended also on the chosen target, we decided to look at the quality of the ensemble of generated samples and to compare it with the conditional distribution used to sample the non-targeted properties. We evaluate this by considering for each target t the set of molecules $\mathcal{S}_t$. For each of these sets, we consider a test distribution $p_t(x) = N(x|\mu_t, \sigma_t)$. The values of μ_t are the ones of the considered targets, while for the values of σ_t we chose a diagonal matrix with diagonal elements $(0.5, 5)$ for targets in the (E_{MBD}, α) plane, and a diagonal matrix with diagonal elements $(0.125, 5)$ for the (ζ, α) plane. The evaluation of the generated samples with respect to their target is then carried out by considering the quantity $\langle -\log(p_t(x)) \rangle_{\mathcal{S}_t}$. The results are reported in Suppl. Fig. 11(b) and Suppl. Fig. 11(d) per each target alongside the relative variances (Suppl. Fig. 11(a) and Suppl. Fig. 11(b)) of the non-targeted properties used in the conditional distributions from the multi-Gaussian fitting (actual variance over the variance of the property across the dataset).

Extended explanation: Figs. 6(a,b) of the main text includes a single structure per target that represents the top 5-molecule set (other structures are depicted in Suppl. Fig. 12). Starting from the target B in (α, E_{MBD})-space (see Fig. 6(a)), one can find molecules with high α and low $|E_{MBD}|$ that show a large and extended highly-conjugated structure with a prevalence of C atoms. Moving from $B \rightarrow A$, we considerably increased $|E_{MBD}|$ while slightly reducing α, generating large and more compact (ring-type) structures with only sigma bonds. This result is consistent with the more sizeable vdW/dispersion energy contributions that arise from the relatively closer non-bonded atoms found within compact atomic arrangements. The 15-

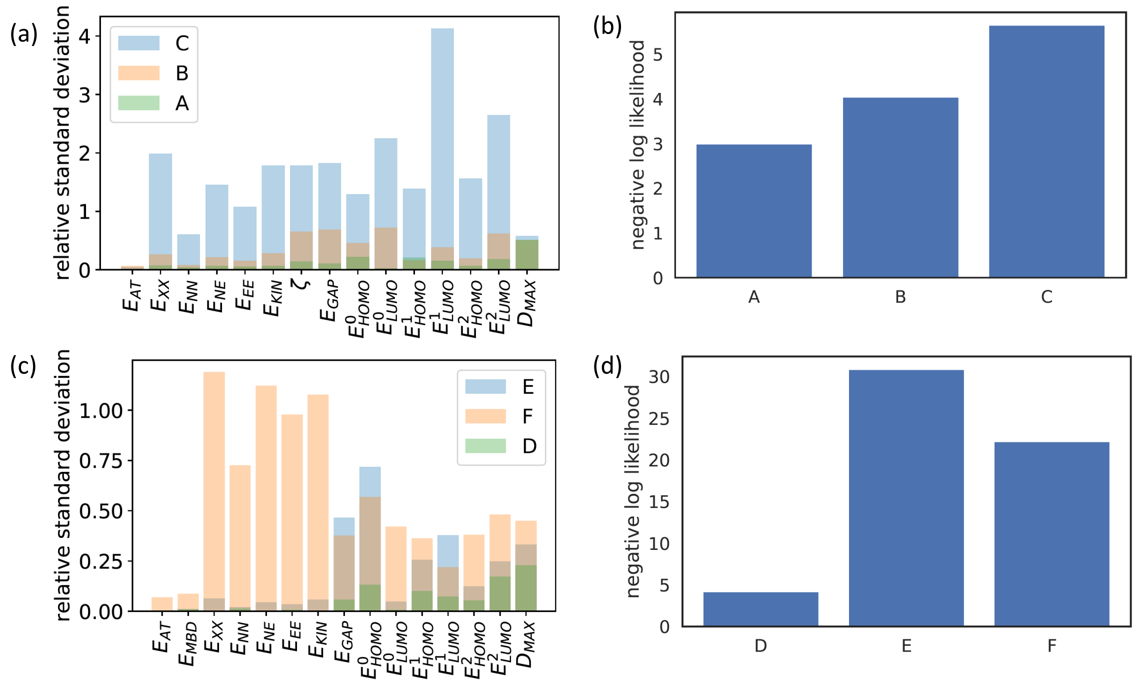

Supplementary Figure 11 (a,c) Relative variance on the non-targeted properties coming from the conditional distributions of the multi-Gaussian fitting. Different colors are used for different targets. (b,d) Average negative log-likelihood of the samples generated per target with respect to a test distribution centered in a given target.

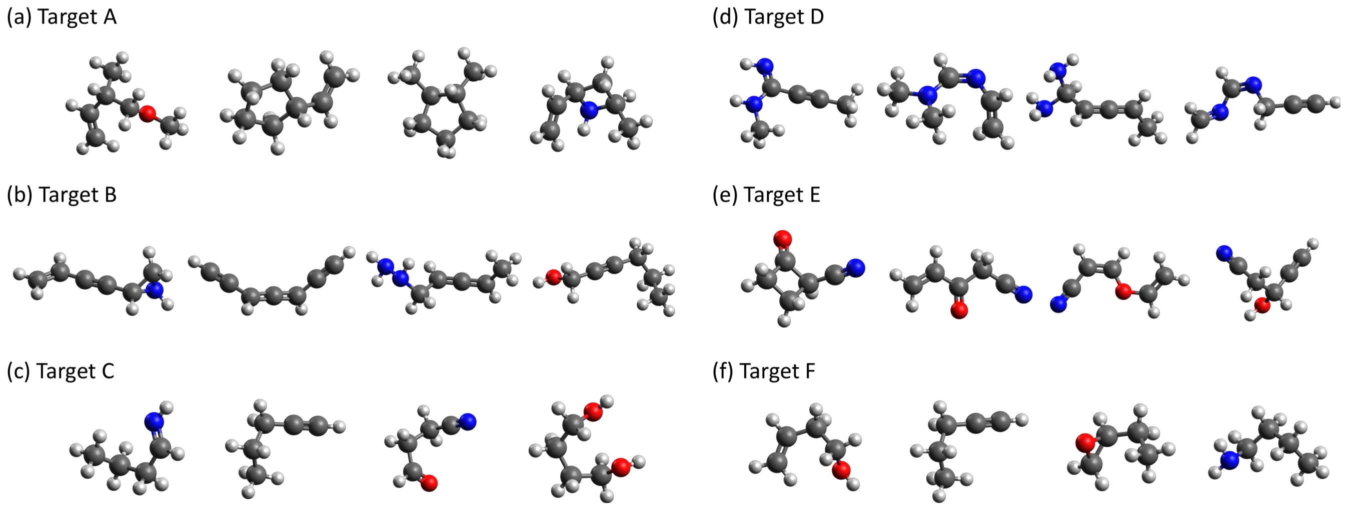

Supplementary Figure 12 Additional molecules generated targeting pair of properties in the 2D property space (a-c) (α, E_{MBD})-space and (d-f) (α, ζ)-space.

molecule set associated to target C (low values of both $|E_{MBD}|$ and α) exhibits a larger diversity compared to molecules in targets A and B, as they consider six distinct heavy atom compositions (such as C_4N, C_4O, C_4NO, C_4O_2, C_5, and C_4) and more complex bonding motifs (*e.g.*, C=O,C≡N, C≡C), which rationalize the differences in the property values when compared to the other target groups. This is a clear example of the flexibility one has when searching for molecules with a desired pair of extensive QM properties. Turning to (α, ζ)-space, the changes in molecular structure resulting from variations in target values can also be understood with physics and chemistry concepts. Thus, in the top 5-molecule set of target D, one can find large and extended C_5N_2 isomers/conformers with high α and moderate values of ζ. By going from $D \rightarrow E$, we obtained slightly more compact structures but with a more complex chemical composition (C_5NO isomers/conformers) mainly yielded by the increment in ζ. A considerable reduction of both properties (target F) generated a top 5-molecule set of structures with fewer heavy atoms and a more homogeneous composition (C_5, C_4O, and C_4N), limiting charge fluctuations.

Comparison with cG-Schnet

To evaluate how well our property-to-structure mapping can work when operating as a conditional generative model, we run additional experiments with the state-of-the-art model developed by Gebauer *et al.*[4] (*i.e.*, cG-Schnet). The choice of this model stems from the fact that, in the original paper, it also presents a two-fold multi-objective task. Accordingly, we trained two cG-Schnet models on conditional generation for the two multi-objective tasks considered in this work, namely (E_{MBD}, α) and (ζ, α). Each model has been trained on the same hardware as the QIM model for a total of four days. Unlike our implementation, cG-Schnet handles H atoms explicitly. In Suppl. Tables 1-4, we compare the performances of our adapted inverse mapping using the same metric defined in the main text We have listed the results for the top 5 and top 15 molecule sets for the targets in the $(E_{\mathrm{MBD}}, \alpha)$ plane and top 5 and top 10 for the targets in the (ζ, α) plane, which were selected as closest in 2D Euclidean distance to the target. Notably, the QIM model presents very close results to those obtained by using cG-Schnet models. Averaging over the targets in each considered group, one can see that the difference in relative error ranges from 0.6% to 5.3% in favor of cG-Schnet model for both molecule sets. The only exception was the prediction of E_{MBD} for the top 15 molecules in the (α, E_{MBD})-space where the difference of ϵ values was $\approx 7.3\%$. This result can be related to the explicit treatment of H atoms during the training of the cG-Schnet models, while in the QIM model H atoms are added after the molecule is generated (see "Methods" section in the main text). Another remark

	Comparison (E_{MBD}, α) top 5 samples			
	E_{MBD}		α	
targets \ models	QIM	cG-Schnet	QIM	cG-Schnet
A	0,46 %	0.91 %	0.40 %	0.60 %
B	4.16 %	1.16 %	2.12 %	0.73 %
C	4.85 %	1.75 %	1.44 %	0.82 %
average	3.16 %	1.27 %	1,32 %	0.71 %

Supplementary Table 1 Comparing performances between the QIM and cG-Schnet models on the conditional generation task defined in the 2D property space (E_{MBD}, α). Here, we report the results averaged over the top 5 generated molecules from both models.

	Comparison (E_{MBD}, α) top 15 samples			
	E_{MBD}		α	
targets \ models	QIM	cG-Schnet	QIM	cG-Schnet
A	6,96 %	1,70 %	2,39 %	0.76 %
B	8,62 %	1,72 %	3,77 %	1,93 %
C	12,16 %	2,32 %	4,46 %	2,26 %
average	9,25 %	1,92 %	3,54 %	1,65 %

Supplementary Table 2 Comparing performances between the QIM and cG-Schnet models on the conditional generation task defined in the 2D property space (E_{MBD}, α). Here, we report the results averaged over the top 15 generated molecules from both models.

	Comparison (ζ, α) top 5 samples			
	ζ		α	
targets \ models	QIM	cG-Schnet	QIM	cG-Schnet
E	6,83 %	4,90 %	2,69 %	0.82 %
F	8,33 %	6,25 %	2.22 %	1,85 %
D	3,55 %	2,43 %	2,37 %	1,43 %
average	6.24 %	4.52 %	2.43 %	1.38 %

Supplementary Table 3 Comparing performances between the QIM and cG-Schnet models on the conditional generation task defined in the 2D property space (ζ, α). Here, we report the results averaged over the top 5 generated molecules from both models.

concerns the possibility of generating molecules beyond 7 heavy atoms. The cG-Schnet model was also able to generate molecules with 8 and 9 heavy atoms for the analyzed targeted pair of QM properties, confirming that atom independence in the molecular representation is a key factor for extrapolation tasks. However, the relative error in property predictions considerably increased reaching values ranging from 5% to 39% for E_{MBD}, 7.2% to 27.7% for α, and 6.2% to 27% for ζ.

models / targets	Comparison (ζ, α) top 10 samples			
	ζ		α	
	QIM	cG-Schnet	QIM	cG-Schnet
E	14,78 %	9,23 %	3,31 %	1.92 %
F	11,53 %	7,30 %	2,62 %	2,43 %
D	9,81 %	3,73 %	3,61 %	1.79 %
average	12,04 %	6.75 %	3,18 %	2,05 %

Supplementary Table 4 Comparing performances between the QIM and cG-Schnet models on the conditional generation task defined in the 2D property space (ζ, α). Here, we report the results averaged over the top 15 generated molecules from both models.

Supplementary references

[1] Dokmanic, I., Parhizkar, R., Ranieri, J. & Vetterli, M. Euclidean distance matrices: Essential theory, algorithms, and applications. *IEEE Signal Process. Mag.* **32**, 12–30 (2015).

[2] Hoffmann, M. & Noé, F. Generating valid euclidean distance matrices (2019). arXiv:1910.03131.

[3] van der Maaten, L. & Hinton, G. Visualizing data using t-sne. *J. Mach. Learn. Res.* **9**, 2579–2605 (2008).

[4] Gebauer, N. W. A., Gastegger, M., Hessmann, S. S. P., Müller, K.-R. & Schütt, K. T. Inverse design of 3d molecular structures with conditional generative neural networks. *Nat. Commun.* **13**, 973 (2022).
